# Experimental evaluation of xenodiagnosis to detect trypanosomes at low parasitaemia levels in infected hosts

**DOI:** 10.1051/parasite/2011184295

**Published:** 2011-11-15

**Authors:** C.M. Wombou Toukam, P. Solano, Z. Bengaly, V. Jamonneau, B. Bucheton

**Affiliations:** 1 Centre international de recherche-développement sur l’élevage en zone subhumide (CIRDES) 01 BP 454 Bobo-Dioulasso 01 Burkina Faso; 2 Institut de recherche pour le développement (IRD), Unité mixte de recherche IRD-CIRAD INTERTRYP, Campus international de Baillarguet Montpellier France

**Keywords:** xenodiagnosis, experimental evaluation, HAT, tsetse, trypanosome, parasitaemia, xénodiagnostic, évaluation expérimentale, THA, glossine, trypanosome, parasitémie

## Abstract

In Human African Trypanosomosis (HAT) endemic areas, there are a number of subjects that are positive to serological tests but in whom trypanosomes are difficult to detect with the available parasitological tests. In most cases and particularly in West Africa, these subjects remain untreated, thus posing a fundamental problem both at the individual level (because of a possible lethal evolution of the disease) and at the epidemiological level (since they are potential reservoirs of trypanosomes). Xenodiagnosis may constitute an alternative for this type of cases. The objective of this study was to update the use of xenodiagnosis to detect trypanosomes in infected host characterized by low parasitaemia levels. This was carried out experimentally by infecting cattle and pigs with *Trypanosoma congolense* and *T. brucei gambiense* respectively, and by feeding tsetse flies (*Glossina morsitans submorsitans* and *G. palpalis gambiensis*, from the CIRDES colonies) on these animals at a time when the observed blood parasitaemia were low or undetectable by the classical microscopic parasitological tests used for the monitoring of infected animals. Our results showed that: i) the *G. p. gambiensis* colony at CIRDES could not be infected with the *T. b. gambiense* stocks used; ii) midgut infections of *G. m. submorsitans* were observed with both *T. congolense* and *T. b. gambiense*; iii) xenodiagnosis remains positive even at very low blood parasitaemia for both *T. congolense* and *T. b. gambiense*; and iv) to implement *T. b. gambiense* xenodiagnosis, batches of 20 *G. m. submorsitans* should be dissected two days after the infective meal. These results constitute a first step toward a possible implementation of xenodiagnosis to better characterize the parasitological status of seropositive individuals and the modalities of parasite transmission in HAT foci.

## Abstract (fr)

En zone d’endémie de la trypanosomose humaine africaine (THA), il existe des suspects positifs en sérologie chez lesquels les trypanosomes sont difficilement détectés en utilisant les tests de diagnostic parasitologique disponibles sur le terrain. Dans la plupart des cas, particulièrement en Afrique de l’Ouest, ces sujets ne sont pas traités, posant ainsi un problème fondamental à la fois au niveau individuel (à cause d’une possible évolution mortelle de la maladie) et au niveau épidémiologique (puisqu’ils sont de potentiels réservoirs de trypanosomes). L’objectif de notre étude était d’actualiser l’utilisation du xénodiagnostic pour détecter les trypanosomes chez des hôtes infectés caractérisés par de faibles parasitémies. Ceci a été fait expérimentalement en infectant des animaux, bovins et porcs, par *Trypanosoma congolense* et *T. brucei gambiense* respectivement, sur lesquels des glossines (*Glossina morsitans submorsitans* et *G. palpalis gambiensis*) de l’élevage du CIRDES étaient nourries lors de parasitémies faibles ou indétectables par des tests parasitologiques microscopiques classiques utilisés pour le suivi des animaux infectés. Nos résultats ont montré que : i) la colonie de *G. p. gambiensis* du CIRDES n’a pas pu être infectée par *T. b. gambiense* ; ii) l’infection de l’intestin moyen des *G. m. submorsitans* a été observée à la fois avec *T. congolense* et *T. b. gambiense* ; iii) le xénodiagnostic demeure positif même à de très faibles parasitémies ; et iv) pour mettre en oeuvre ce xénodiagnostic, des lots de 20 glossines (*G. m. submorsitans*) peuvent être disséqués deux jours après le repas infectant. Ces résultats constituent une première étape vers une possible mise en oeuvre du xénodiagnostic afin de mieux caractériser le statut parasitologique des individus séropositifs et les modalités de transmission du parasite dans les foyers de THA.

## Introduction

Human African Trypanosomosis (HAT) is a parasitic disease caused by two sub-species of trypanosomes: *Trypanosoma brucei* (T. b.) *gambiense* and *T. b. rodhesiense*. *T. b. gambiense*, responsible of 90 % of HAT cases, causes a chronic form of the disease from West to Central sub-Sahara Africa. Parasitism by *T. b. rhodesiense* is associated with a more acute form and is endemic in East sub- Sahara Africa. Trypanosomes are transmitted by tsetse flies (Diptera: Glossinidae). HAT progresses from the hemolymphatic stage, to which no specific clinical symptoms are associated (Jannin *et al.*, 1993), to a meningoencephalitic stage where the parasite is found in the cerebrospinal fluid (CSF) and is associated with the appearance of the neurological clinical signs of the disease. Due to the absence of HAT specific symptoms during the first stage and to the fact that treatment of the meningoencephalitic stage requires the use of highly toxic molecules (melarsoprol causes fatal reactive encephalopathy in 5-10 % of cases), diagnostic is of crucial importance. For these reasons, mass screening of the population is usually performed by the Card Agglutination Test for Trypanosomosis (CATT), and parasitological tests, such as the mini- Anion Exchange Centrifugation Test (m-AECT) or the Buffy Coat Technique (BCT), are then carried out on positive subjects to confirm infection (Jamonneau *et al.*, 2004).

Because in *T. b. gambiense* HAT blood parasitaemia is often very low, the parasitological test used in the frame of medical surveys lack sensitivity. Thus, the parasitological status of a number of subject positive to serological tests but negative in parasitology is currently unknown although increasing data, such as the detection of parasite DNA by different PCR techniques, indicate that at least some of them could be asymptomatic carriers of parasite (Solano *et al.*, 2002; Jamonneau *et al.*, 2004; Koffi *et al.*, 2006; Kaboré *et al.*, 2011). A better parasitological characterization of these seropositive subjects, particularly to know if they can act as a parasite reservoir for tsetse flies is thus of crucial importance to improve control strategies by national control programs. Xenodiagnosis, which consists in detecting the parasite through the dissection of its arthropod vector, after having fed it on patients, is considered as one of the most sensitive parasitological tools, and has already been used in the past for sleeping sickness (Frézil, 1971).

In the present study, our objective was to revisit the possible contribution of xenodiagnosis to HAT diagnosis and to evaluate its sensitivity, by feeding tsetse flies on animals infected experimentally by different trypanosome species at time when blood parasitaemia were low or undetectable by classical parasitological tests.

## Materials and Methods

The experimental protocol received the agreement of the scientific ethics committee of CIRDES for animal care and the use of animals for research purposes.

### Trypanosomes

Two strains of trypanosomes were used in this experiment: a *Trypanosoma congolense* IL 1180 stabilate, originating from Serengeti in Tanzania, and an isolate of *Trypanosoma brucei gambiense* was used in the experimental infection of pig (isolate B4/F303). This isolate of *T. b. gambiense* had been isolated between 2000 and 2004 by rodent inoculation from HAT patients detected in the sleeping sickness focus of Bonon, Côte d’Ivoire, and kept in the cryotheque of IRD/CIRDES. Cryostabilates of these strains were reactivated in mice prior the experimental infections.

### Tsetse Flies

A maximum of 72 hours old teneral males and females *Glossina morsitans submorsitans* and *Glossina palpalis gambiensis* reared at the Centre International de Recherche-Développement sur l’Élevage en Zone Subhumide (CIRDES) insectarium were used. *G. m. submorsitans* flies were fed on infected cows and pig whereas *G. p. gambiensis* flies were fed on infected pig only.

### Animals

Two crossbred zebu cows and one five-month old male pig of local race were used in these experiments. Upon arrival, they were dewormed with Veriben® and treated with oxytetracycline 5 % at recommended dosage. Cows and pig were further kept on different insecticide impregnated net box within a wire-netting surrounding wall for pigs and simple surrounding wall for cows to prevent contact with other flies or other haematophagous insects.

### Experimental Design

#### • Reactivation of stabilates

NMRI mice previously immunosuppressed with cyclophosphamide (Endoxan®, 300 mg/kg), were injected intraperitoneally with 0.3 ml of cryostabilate containing at least 32 × 106 trypanosomes per millilitre (tryp/ml). Immuno-suppression was maintained using cyclophosphamide every five days. The parasitaemia of each mouse was measured daily using the matching method of Herbert & Lumsden (1976).

#### • Experimental infection of animals

Cows: after the reactivation of parasite strains, cows were intraveinously injected with 1 ml of diluted mice blood (in phosphate saline glucose) containing a final concentration of 103 tryp/ml. Following the infection, hematocrite and parasitaemia of cows were monitored daily using the Buffy Coat Technique (BCT) and the parasites counted using the method of Paris *et al.* (1982).

Pig: two weeks after a complete checkup and treatment as previously stated, the pig was immunosuppressed twice with cyclophosphamide (Endoxan®, 300 mg/kg) at three days intervals before infection and twice at three days intervals after infection. Blood parasitaemia was assessed using the BCT before the intravenous and intraperitoneal infection with 0.9 ml of mice infecting blood containing 64 × 106 parasites/ml of the *T. b. gambiense* isolate B4/F303. Following experimental infection, hematocrite and parasitaemia were monitored once every two (or three) days until day (D) 16 using the Buffy Coat Technique (BCT). From D16 onward, additional tests were carried out in addition to BCT: the Card Agglutination Test for Trypanosomiasis (CATT) and the mini-Anion Exchange Centrifugation Test (m-AECT) performed directly on blood (m-AECT-blood) or with an additional concentration step by using buffy coats (m-AECT-buffy-coat) because the parasitological techniques applied so far (BCT and xenodiagnosis with *G. p. gambiensis*) were negative. The sensitivity thresholds of m-AECT-blood and m-AECT-buffy-coat are of 50 and 10 tryp/ml respectively (Buscher *et al.*, 2009; Camara *et al.*, 2010).

#### • Infection of flies

**Fig. 1.**
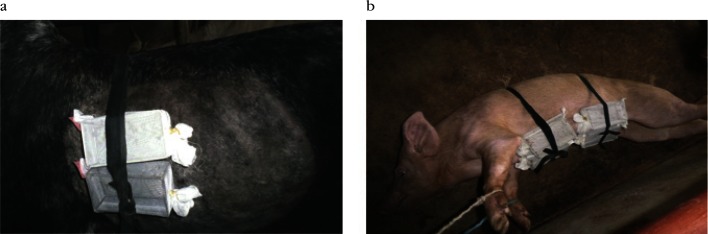
Tsetse flies from the CIRDES colonies fed on a cow (a) and on a pig (b).

On cows: after each parasitological evaluation on infected cow, separate batches of 20 males and females *G. m. submorsitans* were allowed to feed on the side of cows at time when parasitaemia were low (*i.e.* fall under threshold which is not easily detectable by the BCT) or undetectable by the BCT (Fig. 1a). The infecting meal was unique and unfed flies were eliminated. All meals were taken in the obscurity during approximately ten minutes.

On pig: after each parasitological evaluation on infected pig, separate batches of 20 males and females *G. p. gambiensis* and/or *G. m. submorsitans* were allowed to feed every two (or three) days on the abdomen of the infected pigs (Fig. 1b). Only *G. p. gambiensis* was used for that purpose until D16; from D16 onward, both tsetse species were used. All meals were taken in the obscurity during approximately ten minutes as stated for cows and the infective meal was unique. Animals were slaughtered after the experiment.

#### • Dissection of flies

Flies were dissected using the method of Yoni *et al.* (2005). Flies that took their infective meal on cows were dissected at D2 and D5 post infective meal. Those having their infective meal on pig were dissected at D2 post infective meal only. They were dissected after approximately two-three minutes passage at freezing temperature. The midgut was examined by phase-contrast microscope (magnification × 400) to locate and identify infections. Microscopic observation focused on the presence or absence of trypanosomes in the gut. In some cases an estimation of the number of trypanosomes present in the gut was made by examining the entire microscopic preparation.

### Data Analysis

The Fisher’s exact test was used to compare infection rates between males and females at different days post infection.

## Results

### Experimental infections on cows infected with *T. Congolense*

**Fig. 2.**
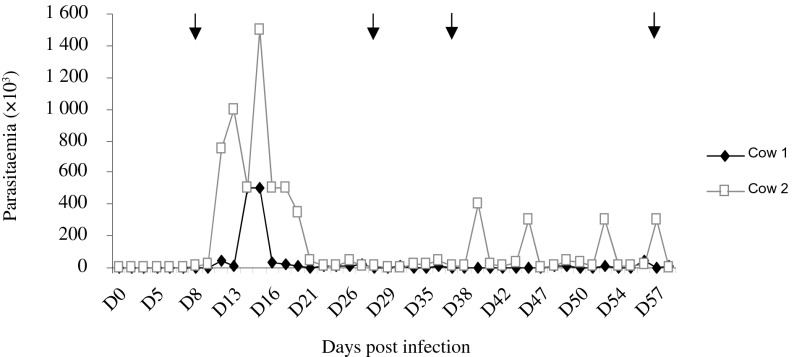
Infection profiles in the cows infected experimentally by *T. congolense*. Arrows indicate the time at which xenodiagnosis was performed with *G. m. submorsitans* on infected animals.

Figure 2 shows the evolution of parasitaemia in cows throughout the experimental period. A peak of parasitaemia was observed at around D16 post infection in both cows. Parasitaemia levels decreased thereafter to low levels in both animals with sporadic peaks observed in one of the cow toward the end of the experiment. This enabled us to feed flies on these animals at different parasitaemia levels.

Infection rates obtained by dissection of flies at two and five days post infective blood meal (PI) are shown in Table I. A total of 372 flies (*G. m. submorsitans*), including 189 females and 183 males were dissected. A total of 52 females and 42 males were found positive of which 50 and 40 were positive at D2 for females and males respectively. The percentage of infected flies at D2 PI is high (> 50 %) when parasitaemia in cows are greater than 1 parasite for 120 microscopic fields.

**Table I.** Results of xenodiagnosis with *G. m. submorsitans* fed on cows infected with *T. congolense* at different parasitaemia.

Blood parasitaemia	2 Tryp 1/field		5 Tryp/40 fields		1 Tryp/80 fields		1 Tryp/120 fields		1 Tryp/160 fields	
DPI 2 flies	D2	D5	D2	D5	D2	D5	D2	D5	D2	D5
NDF^3^	19	18	20	17	18	18	20	20	20	19
NDM 4	19	18	20	20	15	16	20	19	18	18
NPF 5	16	1	19	1	10	0	0	0	5	0
NPM 6	14	0	11	1	12	1	0	0	3	0
% PF 7	84.2	5.6	95.0	5.9	55.6	0.0	0.0	0.0	25.0	0.0
% PM 8	73.7	0.0	55.0	5.0	80.0	6.3	0.0	0.0	16.6	0.0

1 Tryp: trypanosome

2 DPI: days post infective meal

3 NDF : number of dissected female

4 NDM: number of dissected males

5 NPF: number of positive females

6 NPM: number of positive males

7 PF: positive females

8 PM: positive males.

This corresponds to a parasitaemia of 333 tryp/ml according to Paris *et al.* (1982). Below this parasitemic threshold the percentage of infected tsetse becomes lower, though still positive (25 % and 16 % at D2). In contrast, tsetse infection rate is very low or nil at D5 PI whatever the parasitaemia level. At all blood parasitaemia tested, the percentage of infected females at D2 is not different from that of infected males (p > 0.3), except at blood parasitaemia of 5 trypanosomes/40 fields where the percentage of infected females was significantly higher than males (p = 0.008).

### Experimental infection on pig infected with *T. B. Gambiense*

During this experiment, 381 flies (308 *G. p. gambiensis* and 73 *G. m. submorsitans*) took their blood meal on the infected pig. Results of both parasitological tests performed on the animal and infection rates obtained in tsetse flies dissected at D2 PI are shown in Table II. Throughout the experiment no trypanosomes were detected by the BCT, however at D16 the pig was found positive by m-AECT-buffy-coat (one trypanosome in the collector tube) indicating a low parasitaemia in this animal of around 10 tryp/ml which correspond to the sensitivity threshold of the test. Furthermore, the CATT serological test was positive indicating that the animal harbored antibodies directed against trypanosome antigens. At this stage none of the *G. p. gambiensis* flies were found positive by xenodiagnosis. Because we suspected that flies from the *G. p. gambiensis* colony at CIRDES may have somehow lost part of their vectorial competence, we also used *G. m. submorsitans* in further xenodiagnosis experiments. Xenodiagnosis was positive at D19 PI with *G. m. submorsitans* flies (four positive out of 37 dissected) whereas *G. p. gambiensis* flies remained negative. To confirm the difference observed between the two species of tsetse at D19, we repeated the test at D39. At this stage the pig was still negative with the BCT test but remained positive with m-AECTblood (two trypanosomes observed in the collector tube) and m-AECT-buffy-coat (six trypanosomes in the collector tube) indicating that the animal had maintained a low parasitaemia level that can be estimated around 50 and 100 tryp/ml of blood according to the sensitivity thresholds of the tests. In agreement with the result obtained at D19, all *G. p. gambiensis* flies (0/31 dissected) were negative whereas trypanosomes were found in the midgut of three out of the 36 *G. m submorsitans* flies dissected at D2 PI.

**Table II.** Results of xenodiagnosis with *G. m. submorsitans* and *G. p. gambiensis* on the infected pig with *T. b. gambiense*. Results of the serological and parasitological tests performed on the animal are also shown on the right part of the table.

	Xenodiagnosis results										
	Number of dissected flies				Number of infected flies				
	Gpg^2^		Gms^3^		Gpg		Gms		Serological and parasitological diagnosis		
Days PI^1^	F 4	M 5	F	M	F	M	F	M	BCT	CATT	m-AECT
D2-D14	108	105	nd 6	nd	0	0	nd	nd	–	nd	nd
D16	18	16	0	0	0	0	nd	nd	–	++	+
D19	13	17	18	19	0	0	0	4	–	nd	nd
D39	19	12	19	17	0	0	0	3	–	nd	+

1 PI: post infection

2 Gpg: *Glossina palpalis gambiensis*

3 Gms: *Glossina morsitans submorsitans*

4 F: female

5 M: male

6 nd: not done.

### Comparison of Parasitaemia in the Mammalian Host and in the Tsetse Fly

In order to assess the number of trypanosomes present in the infected flies that took their infective blood meal on pig at D39, the whole midgut microscopic preparation was examined and trypanosomes were counted. At least ten trypanosomes were found in each of the infected *G. m. submorsitans* flies. Weighting the flies before and after the blood meal then enabled us to estimate the quantity of blood taken by the flies. We found that flies had taken in average ~ 10 µl of blood. Thus the parasitaemia in the blood meal of infected flies can be estimated to be around 10 trypanosomes per 10 µl of blood, hence 1,000 tryp/ml. This is in deep contrast with our evaluation of the blood parasitaemia in the pig (50-100 tryp/ml, see above).

## Discussion

Xenodiagonosis, in which the natural vector is fed on the patient, or in which infected tissues are injected into a recipient susceptible host, has for long been used to increase the sensitivity of parasite detection in infected hosts. Triatomine bugs have been widely used for the diagnosis of Chagas disease and xenodiagnosis is still considered as the gold standard for parasitological diagnosis (Avilla *et al.*, 1993). Among trypanosomatids, xenodiagnosis was also shown to be efficient for the diagnosis of *Leishmania braziliensis* and *Leishmania infantum* infections (Rojas *et al.*, 1989; Molina *et al.*, 1996). In HAT, some old attempts were also made with tsetse flies to confirm serological suspects. The use of xenodiagnosis was found to be successful in Congo (Frezil, 1971) whereas negative results were obtained in Mali (Challier, 1972, 1973). To our knowledge, no other attempts were made since then, in part because of the challenge of rearing or maintaining tsetse flies in field conditions. Nevertheless experimental studies on cattle infected by *T. congolense* or *T. b. brucei* s.l. have shown that tsetse flies could get infected even in the chronic phase of infection, at times when blood parasitaemia could not be detected by microscopic examination (Mihok *et al.*, 1991; Van Den Bossche *et al.*, 2005; Akoda *et al.*, 2008). Thus data exist suggesting that xenodiagnosis in HAT could represent a highly sensitive tool to detect trypanosomes.

### Experimental Infection in Cattle

In this first experiment we wanted to assess if the tsetse colonies reared at the CIRDES could be used for xenodiagnosis purposes (*i.e.* if they were still able to get infected upon feeding on experimentally infected animals). For this purpose we chose a well known model of cattle infection with *T. congolense* and used teneral *G. m. submorsitans* for the xenodiagnosis procedures. In the two crossbred cows, the infection pattern was characterized by a first pick of parasitaemia (corresponding to the acute phase) followed by a chronic phase in which parasite blood concentrations decreased to sometime very low levels. This situation enabled us to test the efficiency of xenodiagnosis at different parasitaemia levels and to determine at what time post infective blood meal flies should be dissected for xenodiagnosis to be optimum. At high blood parasitaemia levels (≥ 1 trypanosome observed/80 microscopic fields) midgut infection rates were high in both male and female when tsetse flies were dissected at D2 post infective meal. In sharp contrast very few flies were found positive at D5 post infective meal, suggesting that the majority of trypanosomes (at the exception of those able to establish an infection) are killed at that time in the gut, probably by the fly immune system (Gibson & Bailey, 2003; Haines *et al.*, 2010). Anyhow, our results indicate that the optimum day post infective blood meal is D2 because in addition, microscopic examination at D0 or D1 is impaired by the presence of numerous red blood cells in the fly gut (data not shown). The percentage of infected flies at D2 post infective meal decreased sharply below the threshold of 1 trypanosome/ 80 microscopic fields observed in the blood of the animal. However at the lowest parasitaemia tested (1 trypanosome/160 microscopic fields, corresponding to a parasitaemia of 250 tryp/ml of blood), 16,6 and 25 % of *G. m. submorsitans* male and female respectively were still positive (Table I).

These results suggest that at low parasitaemia levels, efficacy seems to be independent of the parasite concentration as reflected by the fact that flies were found infected on cattle harbouring parasitaemia of 1 trypanosome/160 fields but not when they were fed on cattle at 1 trypanosome/120 fields. An alternative explanation is also that at such low parasitaemia level accurate parasite concentration are difficult to obtain with the method of Paris *et al.* (1982). It is noteworthy that in the context of xenodiagnosis, a single infected fly is sufficient to make the test positive. Therefore an adequate number of flies (20 flies, assuming an infection rate of 5 %) are required to maximize the probability to find an infected fly when they are fed on hosts with low blood parasitaemia. Either male or female can be used for this purpose since no difference of infection rates were observed between sexes.

### Experimental Infection in Pigs

In order to further test the sensitivity of xenodiagnosis in conditions that are closer to the situation encountered in the human disease (sleeping sickness or HAT), we then used a pig, a known natural reservoir of *T. b. gambiense* (Mehlitz *et al.*, 1986; Penchenier *et al.*, 2005) that we infected with a field isolate of *T. b. gambiense* recently isolated in West Africa. We then used flies from the *G. p. gambiensis* colony at CIRDES for xenodiagnosis purposes. Interestingly, the pattern of infection in the pig was similar to what is observed in HAT endemic areas on seropositive subjects that are negative to parasitological tests but may sometimes be positive by PCR (Solano *et al.*, 2002; Jamonneau *et al.*, 2004; Koffi *et al.*, 2006; Kaboré *et al.*, 2011).

Throughout the experiment, no trypanosomes could be detected in the pig blood by the BCT technique although we showed that the animal had developed a positive response to the CATT by D16. From this time point we thus chose to use m-AECT, the most sensitive parasitological method (Busher *et al.*, 2009; Camara *et al.*, 2010) to assess the parasitological status of the pig. The pig tested positive to m-AECT on D16 and D39, but with only very few trypanosomes observed in the collector. This indicated that the parasite blood concentration was close to the test detection limit, giving an estimation of the pig parasitaemia in the range of 10-50 and 50-100 tryp/ml of blood at D16 and D39 respectively. Whereas none of the *G. p. gambiensis* flies fed on the animal were found infected throughout the experiment, four out of 37 and three out of 36 flies were found infected when *G. m. submorsitans* was used for xenodiagnosis at D19 and D39 respectively. The observed difference of xenodiagnosis efficiency between *G. m. submorsitans* and *G. p. gambiensis* suggests that the *G. p. gambiensis* colony at CIRDES has somehow lost its vectorial competence, although further experiments will be required to further explore this point. Nevertheless they also indicate that the *G. m. submorsitans* colony at CIRDES can be used for both the xenodiagnosis of *T. congolense* and *T. b. gambiense* infections even at low blood parasitaemia in the infected hosts.

### Differences of parasitemia between tsetse and pigs: co-evolution?

An intriguing observation made in the frame of this study was that the number of trypanosomes present in the gut of *G. m. submorsitans* infected flies was at least ten times higher than the estimated blood parasitaemia. Several hypotheses may explain this observation. A first possibility is that the measured peripheral blood parasitaemia is not representative of the parasitaemia encountered by the tsetse at the skin biting site. An explanation could be that the parasitaemia is higher in capillary blood and/or that additional trypanosomes coming from lymph vessels are present at the biting site. Another hypothesis is that some *Glossina* saliva components, delivered during the blood meal, may act as attractants concentrating trypanosomes at the biting site to increase parasite transmission probability.

Regarding this hypothesis, it is interesting to note that increasing data point out to the fact that tsetse saliva is targeted by trypanosomes to increase their transmission probabilities. Recently, Van Den Abbeele *et al*. (2010) have suggested that trypanosomes have the ability to modify the tsetse saliva composition in a way that alters the fly feeding behaviour to favour parasite transmission. Finally, a last hypothesis is that the few blood stream forms of trypanosomes ingested during the blood meal may undergo a multiplication cycle before entering into procyclic differentiation. This is turn could help the parasite passing through the self cure process that seems to occur in the fly after D2 post infective meal. Further work is now required, in which the blood parasitaemia and the fly parasitaemia throughout the infection process, will need to be measured more accurately to better characterize this aspect of the trypanosome life-cycle. Real time quantitative PCR methods could be used for this purpose.

## Conclusion

These preliminary results indicate that xenodiagnosis of *T. b. gambiense* HAT using the *G. m. submorsitans* colony at CIRDES is technically feasible. According to the results obtained in the frame of this study, we propose the following protocol: i) feeding of a batch of 20 teneral *G. m submorsitans* aged 72 hours; ii) dissection of flies and examination of the midgut two days after the blood meal with an ordinary microscope (magnification × 400). Although the use of xenodiagnosis is unlikely to be widely applicable for HAT diagnosis, it could be of great help to better characterize the parasitological status of unconfirmed serological suspects and to assess their role in transmission. Indeed these subjects constitute a non negligible proportion of the population in endemic areas (Bucheton *et al.*, 2011). Although they are suspected to be infected at very low parasitaemia levels, there is yet no direct evidence that this is indeed the case.
